# Presence of *optrA-*mediated linezolid resistance in multiple lineages and plasmids of *Enterococcus faecalis* revealed by long read sequencing

**DOI:** 10.1099/mic.0.001137

**Published:** 2022-02-07

**Authors:** Martin P. McHugh, Benjamin J. Parcell, Kerry A. Pettigrew, Geoff Toner, Elham Khatamzas, Noha el Sakka, Anne Marie Karcher, Joanna Walker, Robert Weir, Danièle Meunier, Katie L. Hopkins, Neil Woodford, Kate E. Templeton, Stephen H. Gillespie, Matthew T. G. Holden

**Affiliations:** ^1^​ School of Medicine, University of St Andrews, St Andrews, UK; ^2^​ NHS Lothian Infection Service, Royal Infirmary of Edinburgh, Edinburgh, UK; ^3^​ Medical Microbiology, Aberdeen Royal Infirmary, Aberdeen, UK; ^4^​ Medical Microbiology, Forth Valley Royal Hospital, Larbert, UK; ^5^​ Antimicrobial Resistance and Healthcare Associated Infections (AMRHAI) Reference Unit, National Infection Service, Public Health England, London, UK; ^†^​ Present address: Medical Microbiology, Ninewells Hospital, Dundee, UK; ^‡^​ Present address: School of Social and Behavioural Sciences, Erasmus University, Rotterdam, Netherlands; ^§^​ Present address: Department of Medicine III, University Hospital, LMU Munich, Germany

**Keywords:** antimicrobial resistance, *Enterococcus faecalis*, linezolid, *optrA*, plasmid, Tn*6993*

## Abstract

Transferable linezolid resistance due to *optrA*, *poxtA*, *cfr* and *cfr*-like genes is increasingly detected in enterococci associated with animals and humans globally. We aimed to characterize the genetic environment of *optrA* in linezolid-resistant *Enterococcus faecalis* isolates from Scotland. Six linezolid-resistant *E. faecalis* isolated from urogenital samples were confirmed to carry the *optrA* gene by PCR. Short read (Illumina) sequencing showed the isolates were genetically distinct (>13900 core SNPs) and belonged to different MLST sequence types. Plasmid contents were examined using hybrid assembly of short and long read (Oxford Nanopore MinION) sequencing technologies. The *optrA* gene was located on distinct plasmids in each isolate, suggesting that transfer of a single plasmid did not contribute to *optrA* dissemination in this collection. pTM6294-2, BX5936-1 and pWE0438-1 were similar to *optrA*-positive plasmids from China and Japan, while the remaining three plasmids had limited similarity to other published examples. We identified the novel Tn*6993* transposon in pWE0254-1 carrying linezolid (*optrA*), macrolide (*ermB*) and spectinomycin [ANT(9)-Ia] resistance genes. OptrA amino acid sequences differed by 0–20 residues. We report multiple variants of *optrA* on distinct plasmids in diverse strains of *E. faecalis *. It is important to identify the selection pressures driving the emergence and maintenance of resistance against linezolid to retain the clinical utility of this antibiotic.

## Introduction

*Enterococcus faecalis* and *Enterococcus faecium* are carried in the intestinal tract and are important opportunistic pathogens in humans [1]. Treatment of enterococcal infections is challenging due to intrinsic or acquired resistance to multiple antimicrobials including aminoglycosides, benzylpenicillin, cephalosporins, fluoroquinolones, macrolides, tetracyclines and trimethoprim. Among the remaining treatment options, clinical *E. faecium* isolates are usually resistant to amoxicillin, and resistance to vancomycin is increasingly common [2]. In contrast, *E. faecalis* typically remains susceptible to amoxicillin and resistance to vancomycin is uncommon. Where vancomycin cannot be used, treatment options against severe enterococcal infections are largely limited to daptomycin, linezolid or combination therapy and are further complicated by issues with efficacy, susceptibility or toxicity [1].

Oxazolidinones such as linezolid block protein synthesis by binding to the 50S ribosomal subunit and inhibit formation of the initiation complex [3]. Linezolid resistance is reported in ≤1 % of bloodstream enterococcal isolates in the UK and is an important antimicrobial for the treatment of multi-drug-resistant Gram-positive infections, including vancomycin-resistant enterococci [4, 5]. The G2576T mutation in the chromosomal 23S rRNA genes can arise *de novo* during extended linezolid therapy [6], although antimicrobial stewardship and infection prevention and control measures appear to be successful in limiting the generation and spread of mutational linezolid resistance in clinical practice [7]. The methyltransferases Cfr, Cfr(B) and Cfr(D), and the ABC-F ribosomal protection proteins OptrA and PoxtA also confer resistance to linezolid in enterococci but are carried on mobile genetic elements, which can spread across genetically distinct lineages in the absence of antimicrobial selection [8–14]. Recent international surveillance confirmed that linezolid resistance remains rare, but *optrA* has recently spread to every continent and is the dominant mechanism of linezolid resistance in *E. faecalis* [15]. Surveillance has also detected *optrA* in the UK [16]. Studies into the genetic context of *optrA* have identified the gene on both the chromosome and plasmids, often associated with insertion sequences such as IS*1216*, a possible vehicle for the rapid spread of *optrA* [17, 18].

We used whole genome sequencing to determine whether Scottish *optrA-*positive *E. faecalis* isolates represent transmission of a single clonal lineage. We hypothesized that spread of *optrA* is driven by a single mobile genetic element, and to investigate this we made hybrid assemblies of short and long read sequencing data to generate complete genomes and to reconstruct the genetic environment of *optrA*.

## Methods

### Bacterial strains

Study isolates were a convenience sample from three regional hospital laboratories during 2014–17; as such they may not reflect the entire Scottish population of *optrA-*positive *E. faecalis. E. faecalis* were identified from clinical samples using MALDI-TOF MS or the Vitek-2 GP-ID card (bioMérieux). Initial antimicrobial susceptibility testing was performed with the Vitek-2 AST-607 card; where linezolid resistance was detected the full MIC was determined by agar dilution methodology at the AMRHAI reference laboratory, and susceptibility testing was interpreted with EUCAST breakpoints [19]. Linezolid-resistant isolates were then screened for the genetic determinant of resistance at AMRHAI. Detection of the G2576T mutation (* Escherichia coli* numbering) in the 23S rRNA genes was investigated by PCR-RFLP or by a real-time PCR-based allelic discrimination assay [20, 21]. The *cfr* and *optrA* genes were sought by a multiplex PCR using primers for the detection of *cfr* (*cfr-fw*: 5′-TGAAGTATAAAGCAGGTTGGGAGTCA-3′ and *cfr-rev*: 5′-ACCATATAATTGACCACAAGCAGC-3′) [22] and *optrA* (*optrA-F*: 5′-GACCGGTGTCCTCTTTGTCA-3′ and *optrA-R*: 5′-TCAATGGAGTTACGATCGCCT-3′) (AMRHAI, unpublished).

Access to isolates and clinical data was approved by the NHS Scotland Biorepository Network (Ref. TR000126).

### Whole genome sequencing and genomic analysis

Genomic DNA was extracted from pelleted overnight broth cultures using the MasterPure Gram Positive DNA Purification Kit (Cambio), or QiaSymphony DSP DNA Mini Kit (Qiagen). Short read barcoded libraries were prepared using the Nextera XT kit (Illumina) and sequenced with a MiSeq instrument (Illumina) using 250 bp paired-end reads on a 500-cycle v2 kit. Short reads were quality trimmed with Trimmomatic v0.36 and the settings [LEADING:5 TRAILING:5 SLIDINGWINDOW:4 : 15 MINLEN:100] [23]. Barcoded long read libraries were generated with the 1D Ligation Sequencing Kit (Oxford Nanopore Technologies) and sequenced with an R9.4 flow cell on a MinION sequencer (Oxford Nanopore Technologies). Base-calling and barcode de-multiplexing was performed with Albacore v2.1.3 (Oxford Nanopore Technologies) and the resulting fast5 files were converted to fastq with Poretools v0.6.0 [24], or basecalled and de-multiplexed with Albacore v2.3.3 with direct fastq output. Porechop v0.2.3 (https://github.com/rrwick/Porechop) was used to remove chimeric reads and trim adapter sequences. Sequencing reads and annotated assemblies for this study have been deposited in the European Nucleotide Archive at EMBL-EBI under accession number PRJEB36950 (https://www.ebi.ac.uk/ena/data/view/PRJEB36950).

Short reads were mapped to the *E. faecalis* reference genome V583 (accession number AE016830) using SMALT v0.7.4 [25]. Mapped assemblies were aligned, and regions annotated as mobile genetic elements in the V583 genome (transposons, integrases, plasmids, phages, insertion sequences, resolvases and recombinases) were removed from the assembly (https://github.com/sanger-pathogens/remove_blocks_from_aln). All sites in the alignment with SNPs were extracted using SNP-sites v2.4.0 [26] and pairwise SNP counts were calculated (https://github.com/simonrharris/pairwise_difference_count).

MLST profiling was performed using SRST2 v0.2.0 [27] and the *E. faecalis* MLST database (https://pubmlst.org/efaecalis/) sited at the University of Oxford [28, 29]. Antimicrobial resistance mechanisms were detected using ARIBA v2.12.1 [30] and the ResFinder database v3.0 [31] with the addition of linezolid resistance mutations in the 23S rRNA (G2505A and G2576T based on *E. coli* numbering) and *rplC*, *rplD*, and *rplV* ribosomal protein genes.

Hybrid assembly was performed with Illumina short reads and Nanopore long reads using Unicycler v0.4.7 [32] in standard mode. The resulting assemblies were annotated with Prokka v1.5.1 using a genus-specific RefSeq database [33]. Hybrid assemblies were checked for indel errors using Ideel (https://github.com/mw55309/ideel) and UniProtKB TrEMBL database v2019_1. Plasmid comparisons were generated and visualized with EasyFig v2.2.2 [34].

## Results

### Detection of *optrA* in distinct *E. faecalis* strains

There were 14133 isolates of *E. faecalis* during the study period from all sample types: 14 (0.1 %) were identified as linezolid-resistant, and eight (57.1%) were confirmed as *optrA*-positive at the AMRHAI reference laboratory. Six *optrA*-positive *E. faecalis* were available for further characterization (Table 1). The earliest isolates in this collection were from the Grampian region in the northeast of Scotland in 2014, 2015 and 2016. Three more isolates were identified in 2017 from the Lothian and Forth Valley regions in east and central Scotland (Table 1), with no clear epidemiological links between the patients. Only one patient had known exposure to linezolid prior to the isolation of an *optrA*-positive *E. faecalis *, two patients were hospitalized at the time of sample collection while the remaining four were from general practice. Samples were collected for symptomatic urinary tract infection or orchitis.

**Table 1.** Details of the *optrA*-positive *E. faecalis* characterized in this study

Isolate	Year	Region	Clinical sample	Patient source	MLST	Acquired linezolid resistance genes					Mutations in 23S rRNA		Mutations in ribosomal proteins*			MIC (mg l^–1^)	
						*cfr*	*cfr*(B)	*cfr*(D)	o*ptrA*	*poxtA*	G2505A	G2576T	L3	L4	L22	CHL	LZD
WE0851	2014	Grampian	Urine	GP	480	−	−	−	+	−	−	−	T150A	F101L	−	≥64	8
WE0254	2015	Grampian	Urine	GP	19	−	−	−	+	−	−	−	T150A	F101L	−	≥64	8
WE0438	2016	Grampian	Urine	Hospital	330	−	−	−	+	−	−	−	T150A	F101L	−	≥64	8
TM6294	2017	Forth Valley	Urine	Hospital	585	−	−	−	+	−	−	−	T150A	F101L	−	≥64	8
BX5936	2017	Lothian	Semen	GP	894	−	−	−	+	−	−	−	T150A	F101L	−	≥64	8
BX8117	2017	Lothian	Urine	GP	16	−	−	+	+	−	−	−	T150A	F101L	−	≥64	8

GP, general practice.

*The mutations identified here have never been detected in the absence of other resistance mechanisms in linezolid-resistant isolates, and have been detected in linezolid-susceptible isolates. Their role in linezolid resistance is unclear [54].

Whole genome sequencing was performed to investigate the genetic relationship between the isolates. *In silico* MLST showed the six isolates belonged to different STs, suggesting they were genetically distinct (Table 1). To further confirm this, we analysed SNPs in the core genomes of the *optrA*-positive isolates and found the isolates differed by a median 18806 SNPs (range 13 909–22 272). Previous estimates suggest a genetic diversification rate of 2.5–3.4 SNPs/year for *E. faecalis *, highlighting the *optrA*-positive isolates share a very distant common ancestor [35].

### *optrA* is carried on diverse genetic platforms

Hybrid assembly produced complete or near-complete genomes with <3 % putative coding sequences shorter than the closest reference match. This indicated the hybrid assembly process removed most indel errors, with 1–5 % of coding sequences expected to represent true truncated pseudogenes [36]. The hybrid assemblies contained between one and three plasmids ranging in size from 11 to 80 kb, with *optrA* present on a single complete plasmid in each isolate (pBX5936-1, pBX8117-2, pTM6294-2, pWE0254-1, pWE0438, pWE0851-1; Table S1, available in the online version of this article).

**Fig. 1.**
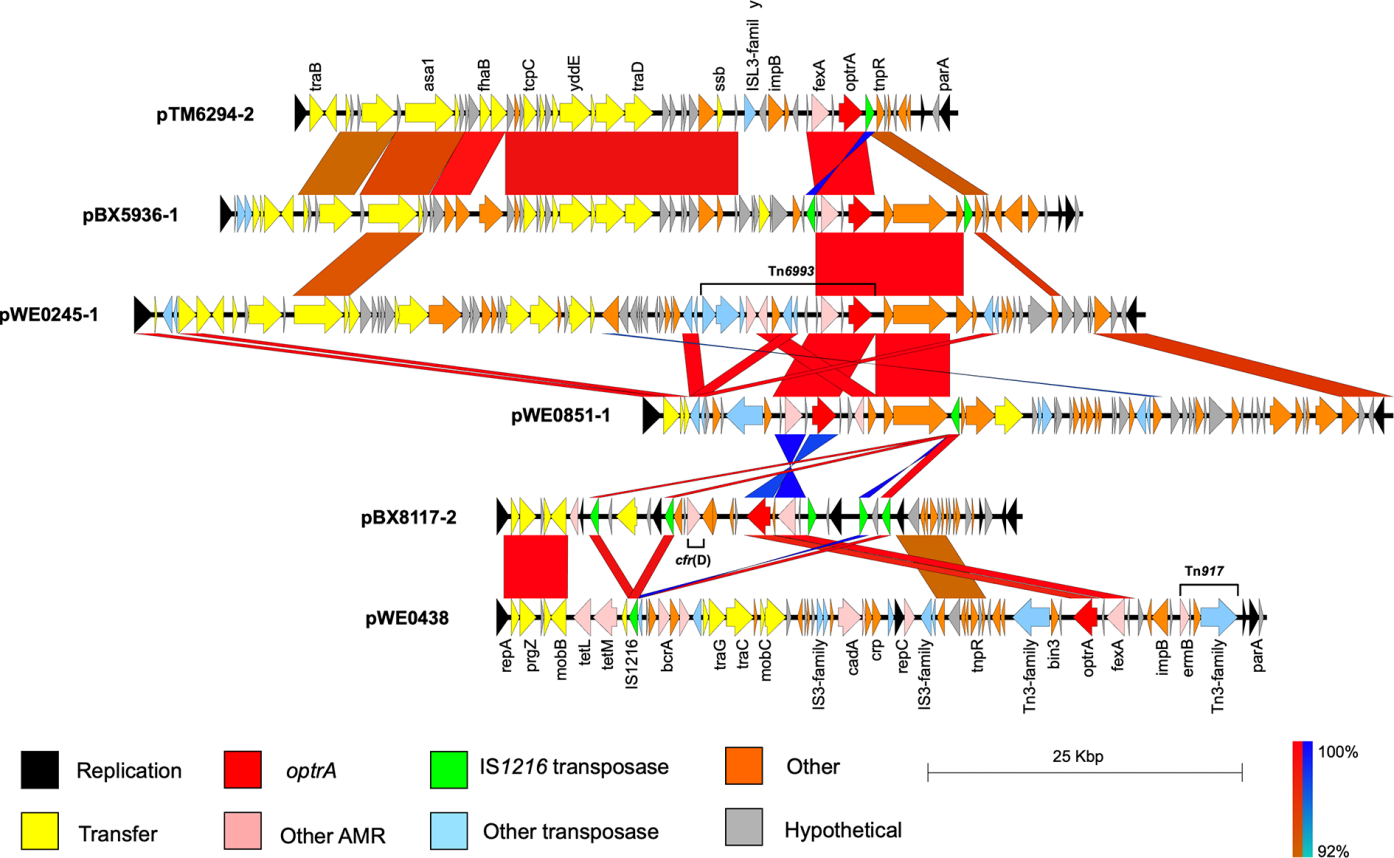
Alignment of full *optrA*-positive plasmid sequences. While some sequence similarity is seen between pTM6294-2 and pBX5936-1, in general identity is low between the *optrA*-positive plasmids, indicating *optrA* has mobilized to multiple plasmid backbones. Arrows indicate coding sequences, coloured blocks between each sequence indicate regions with blastn sequence identity ≥90 % and length >680 bp. Blue identity blocks indicate inverted sequence.

The *optrA*-positive plasmids shared limited sequence similarity to the first described *optrA* plasmid (pE394, accession KP399637), with only the 5–10 kb region surrounding *optrA* and *fexA* (a chloramphenicol/florfenicol exporter) showing >70 % nucleotide identity. In all six Scottish *optrA-*positive plasmids, *optrA* and *fexA* were located within 550–750 nt of each other intervened by a single coding sequence (hypothetical function in all but pBX8117-2 which was annotated as a putative NADH reductase). Within the Scottish *optrA*-positive plasmids, pBX5936-1 (69 kb) and pTM6294-2 (53 kb) were most similar, sharing 97 % average nucleotide identity over 40 kb of aligned sequence (Fig. 1). pTM6294-2 shared 99.8 % identity with a 53 kb *optrA*-positive pheromone responsive plasmid detected in *E. faecalis* from a clinical sample in China (pEF10748), clinical samples in Spain (IsoBar1, IsoBar2 and IsoBar3) and raw dog food in Portugal (pAPT110) [37, 38]. pWE0438 shared 92.3 % nucleotide identity over 52 kb with pS7316 from an *E. faecalis* isolated from a hospitalized patient in Japan [39]. In pWE0438, the *optrA* and *fexA* genes were ~3.8 kb upstream of Tn*917* carrying *ermB*, and ~1.8 kb downstream of another Tn*3*-family transposase (Fig. 1). pBX8117-2 carried *optrA* and the novel *cfr*(D) gene (encoding a 23S rRNA methylase that confers phenicol, oxazolidinone, pleuromutilin and streprogramin A resistance) but apart from these genes showed no similarity to another *E. faecium optrA*/*cfr*(D)-positive plasmid identified in a clinical sample in Ireland (M17-0314) [40]. The other Scottish *optrA-*positive plasmids showed limited similarity to other published examples outside of the *optrA*/*fexA* region.

IS*1216* is often associated with *optrA* and other antimicrobial resistance genes in enterococci. pBX5936-1 and pBX8117-2 had IS*1216* flanking the *optrA* and *fexA* region as a putative transposable cassette (Figs 1 and S1). However, IS*1216* can mobilize from a single insertion sequence copy [41] and single copies were found close to *optrA* in pTM6294-2 and pWE0851-1 (Figs 1 and S1). blastn comparison of pWE0254-1 with the other *optrA*-positive plasmids highlighted a partial IS*1216* transposase that was not identified by automated annotation. Immediately upstream of the partial IS*1216* was an IS*3*-family transposase, the insertion of which probably disrupted IS*1216*. In pWE0254-1 *optrA* and *fexA* were found on a Tn*6674*-like element carrying macrolide (*ermA*) and spectinomycin (APH(9)-Ia) resistance genes. The element was 98.9 % identical to Tn*6674* but had a 1.2 kb insertion containing IS*3*-family transposases (Fig. S1), and was classified as Tn*6993* by the Transposon Registry (accession GCA_906464915) [42]. Tn*6993* was not inserted into the chromosomal *radC* gene as described for most Tn*6674*-like elements [43, 44]. A similar element was present in a plasmid from *E. faecalis* in Chinese swine (TBCP-4814-p1, accession MH830363) but this element lacked the *tnpA* gene and the 1.2 kb insertion of Tn*6993* (Fig. S1) [45]. pWE0438 had a single copy of IS*1216* located ~35 kb from *optrA*, although Tn*917* and Tn*3*-like transposases were detected closer to *optrA* as described above.

### *optrA* sequences vary between isolates

Comparison of the *optrA* sequence from each isolate to the first identified *optrA* from pE394 revealed different variants at the nucleotide and amino acid levels: WE0254 and TM6294 had one synonymous nucleotide substitution, BX5936 had a single non-synonymous nucleotide substitution, WE0851 had two non-synonymous nucleotide substitutions, WE0348 had three non-synonymous and one synonymous substitution, and BX8117 had 20 non-synonymous and a further 17 synonymous substitutions (Table S1, Fig. S2). The degree of sequence variation between the six FexA proteins was less than that seen in OptrA. Comparison with the first reported FexA sequence (AJ549214) showed four common non-synonymous variants in all strains (amino acid changes A34S, L39S, I131V and V305I), with all but BX8117 having an additional D50A variant.

## Discussion

This study found *optrA* present in diverse genetic lineages of *E. faecalis* and carried on largely unrelated plasmids in six isolates from Scotland. pTM6294-2, pBX5936-1 and pWE0438 shared homology with plasmids identified in China or Japan, highlighting the wide dispersal of *optrA*. However, the other Scottish plasmids had limited similarity to other published examples, suggesting a diverse reservoir of *optrA*-carrying genetic elements. We identified *optrA* often carried with a number of other resistance genes, including in a novel multiresistance transposon Tn*6993* in pWE0254-1, and the recently described *cfr*(D) in pBX8117-2. Despite differences in *optrA* sequences and carriage of other linezolid determinants such as *cfr*(D), all isolates showed low-level linezolid resistance of 8 mg l^−1^ (Table 1).

Freitas *et al*. [44] recently analysed all publicly available *optrA*-positive genome sequences and categorized the genetic environment of *optrA*. Group 1 includes Tn*6674*-like platforms, of which WE0254 is a representative (Fig. S1). However, in the original scheme all Group 1 elements were integrated into the chromosome, while in WE0254 the *optrA* element Tn*6993* is inserted into a plasmid. Group 2 includes *optrA-fexA-impB* platforms, represented in the Scottish isolates by TM6294 and WE0438 (Fig. S1). Group 3 includes platforms containing the *araC* regulatory element and is not represented in the Scottish *optrA*-positive isolates characterized here. The three remaining Scottish isolates could not be grouped based on the Freitas scheme, highlighting the need for further studies and public access to complete genome sequences to determine the true diversity of *optrA*-positive platforms.

Many studies of *optrA* to date are from China and tend to show a higher prevalence of *optrA* in isolates from animals rather than humans [11, 46, 47]. Additionally, florfenicol use in agriculture is linked to *optrA* detection in farm animals [48, 49]. However, increasing reports describe rapid increases in *optrA* detection from human samples in many countries [15, 50, 51]. *optrA*-positive isolates are often resistant to multiple antibiotic classes used in animal and human health, allowing significant opportunity for co-selection of *optrA*-positive strains both in animal and in human settings. More recently, *optrA* has been identified in clinical vancomycin-resistant *E. faecium* isolates, with very limited treatment options [50, 52, 53].

Our study is limited in scale as we only include isolates from three regional clinical laboratories, and therefore larger studies are required to infer national patterns. However, our finding that *optrA* is present as different gene variants, carried on different mobile genetic elements, in unrelated strains of *E. faecalis* suggest a diverse *optrA* reservoir that is only partly investigated in this study.

As well as *optrA*, the *cfr* and *poxtA* genes are emerging transferable linezolid resistance mechanisms. Further studies from a One Health perspective are warranted to understand the selection pressures driving transferable linezolid resistance, and the transmission dynamics of these strains to avoid further spread of oxazolidinone resistance within *E. faecalis* and other Gram-positive bacteria.

## Supplementary Data

Supplementary material 1

Supplementary material 2
