# Supplementary material for: Presence of optrA-mediated linezolid resistance in multiple lineages and plasmids of Enterococcus faecalis revealed by long read sequencing
Source: Microbiology (Reading). 2022 Feb 7;168(2):001137. doi: 10.1099/mic.0.001137 (PMC8941993; doi:10.1099/mic.0.001137)
Supplement: Supplementary material 1 [file mic-168-1137-s001.pdf]

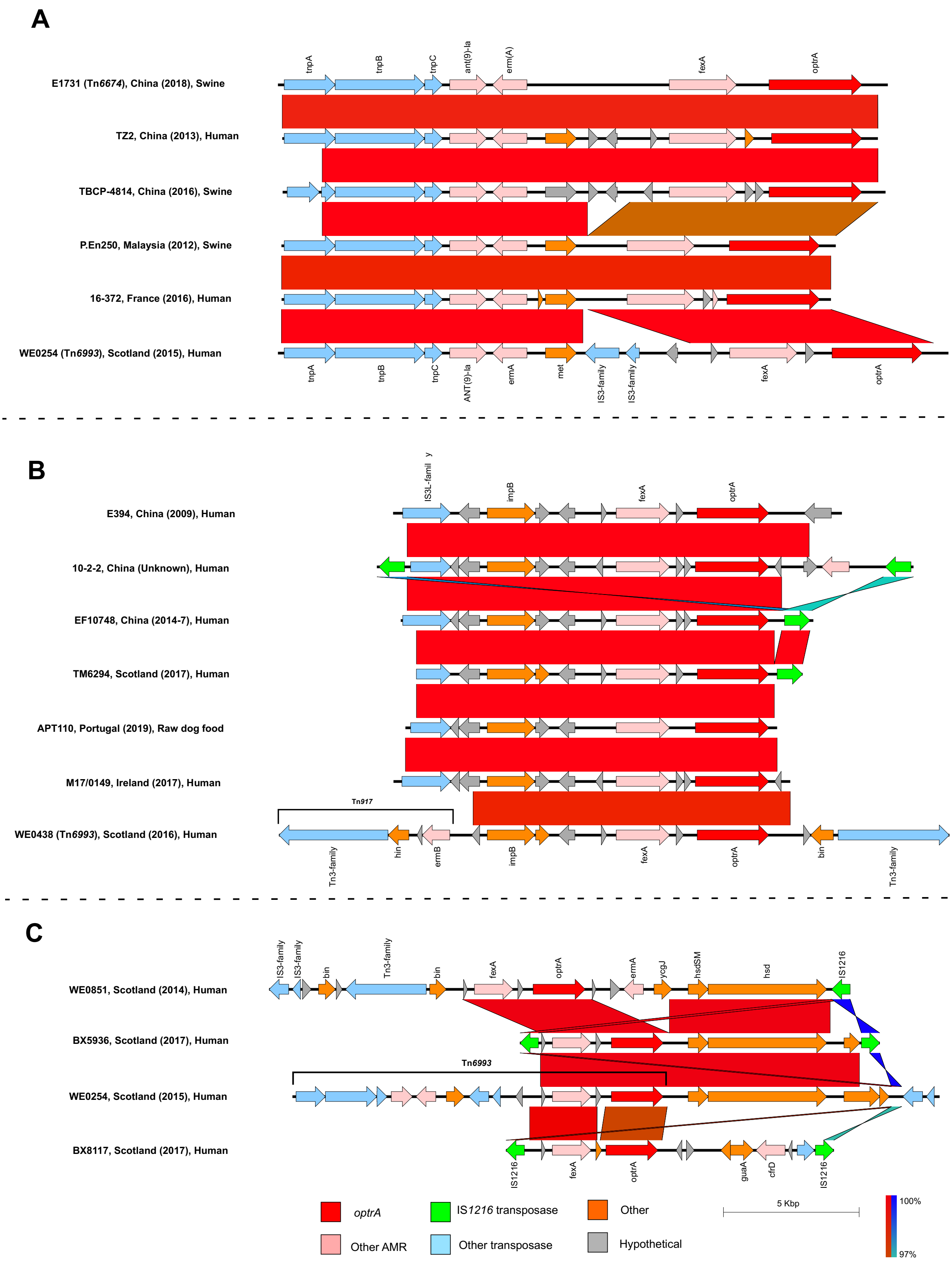

Supplementary Figure 1. Examples of different platforms carrying the *optrA* gene from diverse sources. Panel A includes examples of Tn*6674*-like platforms or Group 1 according to Freitas et al (2020); panel B includes *impB-fexA-optrA* platforms or Group 2; panel C includes the three isolates from this study that do not fit in the Freitas groupings, as well as WE0254 for comparison as it has conserved

hsd/hsdSM restriction enzyme/methylase genes with WE0851 and BX5936 which may have a role in element maintenance. Arrows indicate coding sequences, coloured blocks between each sequence indicate regions with >97% BLASTn sequence identity length >300bp. Labels indicate the ID, country, year and source of isolate.

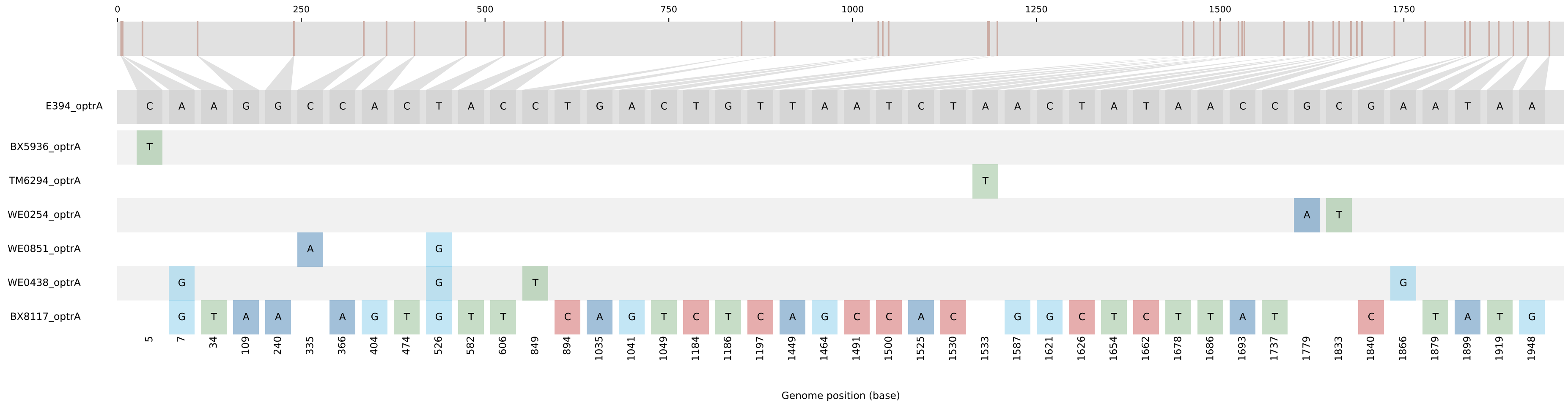

Supplementary Figure 2. Nucleotide variants detected in Scottish *optrA* sequences. Variants against the first identified *optrA* identified in an *E. faecalis* isolated from a clinical sample in China in 2009 (pE394, accession KP399637). Figure made with Snipit (https://github.com/aineniamh/snipit)
